# Ambient air pollution and years of life lost in Ningbo, China

**DOI:** 10.1038/srep22485

**Published:** 2016-03-01

**Authors:** Tianfeng He, Zuyao Yang, Tao Liu, Yueping Shen, Xiaohong Fu, Xujun Qian, Yuelun Zhang, Yong Wang, Zhiwei Xu, Shankuan Zhu, Chen Mao, Guozhang Xu, Jinling Tang

**Affiliations:** 1 Ningbo Municipal Center for Disease Control and Prevention, Ningbo, China; 2 Division of Epidemiology, JC School of Public Health and Primary Care, The Chinese University of Hong Kong, Hong Kong; 3 Guangdong Provincial Institute of Public Health, Guangdong Provincial Center for Disease Control and Prevention, Guangzhou, China; 4 School of Public Health, Soochow University, Suzhou, China; 5 School of Public Health and Social Work, Queensland University of Technology, Brisbane, Australia; 6 Injury Control Research Center, Zhejiang University School of Public Health, Hangzhou, China; 7 Shenzhen Key Laboratory for Health Risk Analysis, Shenzhen Research Institute of The Chinese University of Hong Kong, Shenzhen, China

## Abstract

To evaluate the burden of air pollution on years of life lost (YLL) in addition to mortality, we conducted a time series analysis based on the data on air pollution, meteorological conditions and 163,704 non-accidental deaths of Ningbo, China, 2009–2013. The mean concentrations of particulate matter with aerodynamic diameter <10 μm, particulate matter with aerodynamic diameter <2.5 μm, sulfur dioxide and nitrogen dioxide were 84.0 μg/m^3^, 60.1 μg/m^3^, 25.1 μg/m^3^ and 41.7 μg/m^3^, respectively. An increase of 10-μg/m^3^ in particulate matter with aerodynamic diameter <10 μm, particulate matter with aerodynamic diameter <2.5 μm, sulfur dioxide and nitrogen dioxide was associated with 4.27 (95% confidence interval [CI] 1.17–7.38), 2.97 (95% CI −2.01–7.95), 29.98 (95% CI 19.21–40.76) and 16.58 (95% CI 8.19–24.97) YLL, respectively, and 0.53% (95% CI 0.29–0.76%), 0.57% (95% CI 0.20–0.95%), 2.89% (95% CI 2.04–3.76%), and 1.65% (95% CI 1.01–2.30%) increase of daily death counts, respectively. The impact of air pollution lasted for four days (lag 0–3), and were more significant in the elderly than in the young population for both outcomes. These findings clarify the burden of air pollution on YLL and highlight the importance and urgency of air pollution control in China.

Along with its rapid economic growth and the accompanying urbanization and industrialization in the past decades, China is faced with the problem of serious ambient air pollution caused by increasing consumption of coal, power plant emissions, vehicle exhaust and dust from construction sites^1^. The adverse impact of air pollution on health has become a great public concern. Air pollution is a complex, heterogeneous mixture of gaseous pollutants and particulate matter (PM) that may vary in composition with geographical areas and meteorological conditions^2^. The gaseous compounds causing air pollution mainly include sulfur dioxide (SO_2_), nitrogen oxides, ozone, and carbon monoxide^2^,^3^. PM is usually defined according to its aerodynamic diameter, and the PM with an aerodynamic diameter <10 μm (PM_10_) is of great concern, because such pollutants, once inhaled, could ultimately enter the lungs of human and cause serious problems of lungs and heart^2^,^3^. PM_10_ is further divided into coarse (2.5 to 10 μm; PM_2.5–10_), fine (<2.5 μm; PM_2.5_), and ultrafine (<0.1 μm; PM_0.1_) particles, with smaller particles generally being more deadly in terms of their health effects. Currently, PM_10_, SO_2_ and nitrogen dioxide (NO_2_) are the principal air pollutants in China^4^, and PM_2.5_ has become a hot topic due to the heavy smog frequently seen in many large cities such as Beijing.

A large body of evidence has linked air pollution with excess morbidity and mortality^2^,^5^,^6^,^7^,^8^,^9^,^10^,^11^,^12^. The majority of previous studies that examined the impact of air pollution on excess mortality used daily death counts as dependent variable in their regression analyses. This approach focuses on number of deaths and ignores the difference in ages of deaths, implicitly giving equal weights to the deaths occurring at a young age and those occurring at an old age^13^. However, from a public health perspective, deaths occurring at different ages are not equally important, as the life expectancy of young people is longer than that of elderly people, which means that dying at a young age results in more potential years of life lost (YLL)^13^. In this sense, YLL is more accurate than daily death counts to represent premature deaths and could be an important complementary index to measure excess mortality^14^,^15^.

To date, however, studies using YLL as outcome to assess air pollution impact on human health have been sparse^16^, mainly due to the lack of individual data needed for calculating YLL. In addition, the relationship of air pollution with YLL may vary geographically for multiple reasons^17^,^18^,^19^. To facilitate evidence-based policy-making and resource allocation, this study further evaluate the impact of air pollution on daily YLL in addition to daily death counts based on a large individual dataset of Ningbo, China.

## Results

**Figure 1:**
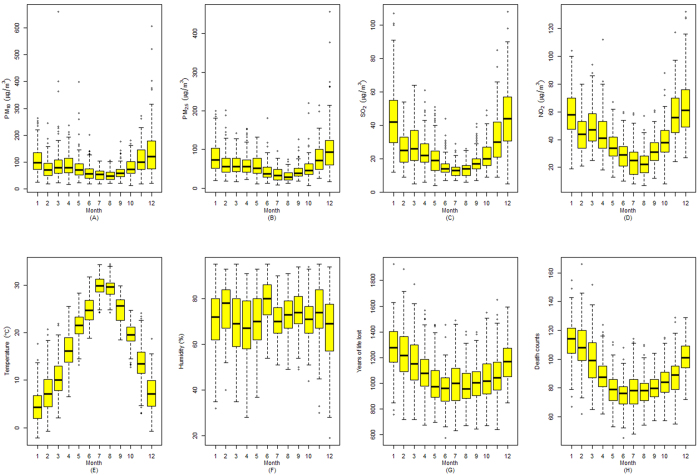
Boxplots of monthly PM_10_, PM_2.5_, SO_2_, NO_2_, temperature, relative humidity, years of life lost and death counts in Ningbo, China, 2009–2013.

Summary statistics of daily air pollutants, meteorological conditions, deaths count, and YLL are listed in Table 1. The mean concentrations of PM_10_, PM_2.5_, SO_2_ and NO_2_ were 84.0 μg/m^3^, 60.1 μg/m^3^, 25.1 μg/m^3^ and 41.7 μg/m^3^, respectively, compared with the World Health Organization air quality guideline levels of 20 μg/m^3^, 10 μg/m^3^, 20 μg/m^3^ and 40 μg/m^3^, respectively^3^. Different air pollutants and meteorological parameters were correlated with each other, with the most significant correlation observed between the four pollutants (Table 2). Daily death counts and YLL had a mean of 89.7 and 1078 years, respectively. Both outcomes showed a seasonal trend, with higher values in November through the next April than in other months (Fig. 1).

**Figure 2:**
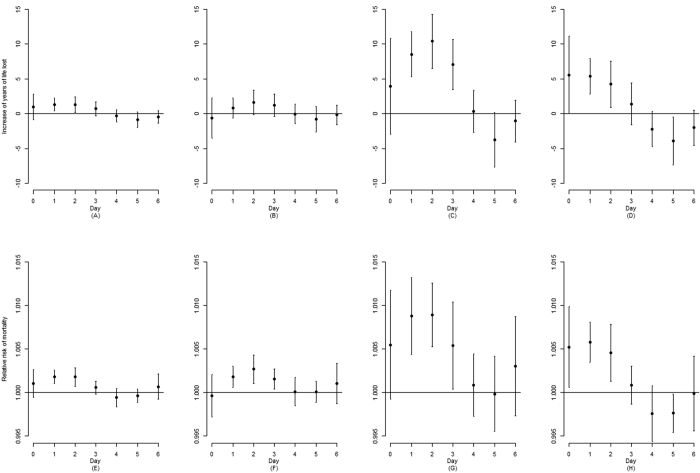
Association of a 10-μg/m^3^ increase of PM_10_ (**A,E**), PM_2.5_ (**B,F**), SO_2_ (**C,G**), and NO_2_ (**D,H**) with increase of years of life lost and relative risk of mortality using single-pollutant models at different lag days in Ningbo, China, 2009–2013, adjusting for seasonality, day of the week, temperature, relative humidity, air pressure and wind speed. The figures for PM_2.5_, i.e. (**B,F**), were based on data collected from 2011 to 2013 only.

**Figure 3:**
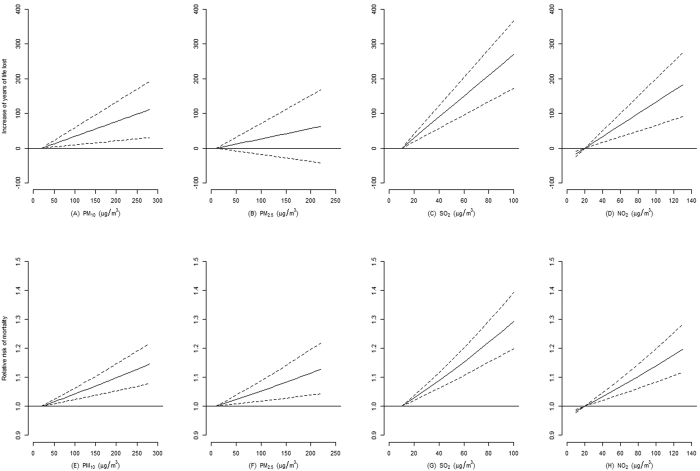
Association of air pollutants (lag 0–3 day) with increase of years of life lost and relative risk of mortality in Ningbo, China, 2009–2013. A natural cubic spline with five degrees of freedom for lag structure was included in the single-pollutant models, adjusting for seasonality, day of the week, temperature, relative humidity, air pressure and wind speed. The figures for PM_2.5_ were based on data collected from 2011 to 2013 only.

The association with air pollutants lasted for four days (lag 0–3), and the lag patterns were similar for YLL and daily death counts (Fig. 2). The four-day cumulative association with an increase of 10-μg/m^3^ in pollutants on YLL and daily death counts were summarized in Table 3 and graphically presented in Fig. 3. Briefly, in single-pollutant model, an increase of 10-μg/m^3^ in PM_10_, PM_2.5_, SO_2_ and NO_2_ was associated with 4.27 (95% confidence interval [CI] 1.17–7.38), 2.97 (95% CI −2.01–7.95), 29.98 (95% CI 19.21–40.76) and 16.58 (95% CI 8.19–24.97) YLL, respectively, and 0.53% (95% CI 0.29–0.76%), 0.57% (95% CI 0.20–0.95%), 2.89% (95% CI 2.04–3.76%), and 1.65% (95% CI 1.01–2.30%) increase in daily death counts, respectively. In two- and three-pollutant models, the estimates for associations with PM (PM_10_ and PM_2.5_) decreased dramatically when gaseous pollutants (SO_2_ and NO_2_), SO_2_ in particular, were added to the model. The estimates for associations with NO_2_ also decreased when SO_2_ was added. The inclusion of PM_10_, PM_2.5_ or NO_2_ into the model did not influence the estimates for association with SO_2_ much.

As data for PM_2.5_ were available from 2011 to 2013 only, we conducted sensitivity analyses for the results in Table 3 by using data of 2011–2013 alone. It was found that the relative magnitude of effects of different pollutants and their changes in two- or three-pollutant models were of similar pattern to those in Table 3 (Supplementary Table S1). Additional sensitivity analyses were conducted by changing the degrees of freedom for per year of time from 6 to 8, which did not materially alter the results of Table 3 either (Supplementary Table S2).

The results of subgroup analyses by sex, age and cause of death are summarized in Table 4. Although the estimates of association across subgroups overlapped a lot in 95% CIs and were not statistically significantly different possibly due to insufficient statistical power, there was a trend that the estimates for association with gaseous pollutants (SO_2_ and NO_2_) were stronger in females, the elderly and those with cardiovascular diseases (for YLL) or those with respiratory diseases (for daily death counts), while the estimates for association with particulate matter (PM_10_ and PM_2.5_) were stronger in males, the elderly and those with respiratory diseases, regardless of the outcome. We also conducted stratified analyses to investigate the modifying effects of temperature and humidity on the associations between pollutants and outcomes (Table 5). The relative strength of associations with different pollutants was, in most scenarios, consistent with the results of total analyses. However, the association was much stronger in days with “high temperature and low humidity” than in others, regardless of pollutants and outcomes, suggesting that temperature and humidity had modifying effects.

## Discussion

While the association of air pollution with morbidity and mortality risk is well documented, few studies have examined the impact of air pollution on YLL. The present study based on data of 163,704 non-accidental deaths over a five-year period found that PM_10_, PM_2.5_, SO_2_ and NO_2_ were all associated with YLL. This is consistent with a previous study conducted in the urban districts of Beijing, China, 2004–2008^16^, which, to our knowledge, was the only study from China looking at YLL. Our analysis demonstrated a general trend that the associations of air pollution with daily death counts and YLL were both stronger in the elderly than in younger people. This is somewhat different from the findings of Guo *et al.* Specifically, in their study, Guo *et al.* found that the association of air pollution with daily death count was stronger in the elderly (>65 years) than in those aged ≤65 years, whereas the association with daily YLL was stronger in younger people. As daily total YLL is determined by daily death counts and YLL of individual death cases, the results of Guo *et al.* imply that YLL of young death cases in Beijing was significantly affected by the high-level air pollution (mean concentrations of PM_10_, PM_2.5_, SO_2_ and NO_2_ were 144.6, 105.1, 48.6, and 64.2 μg/m^3^ respectively), while in Ningbo the relatively low-level air pollution (mean concentrations of PM_10_, PM_2.5_, SO_2_ and NO_2_ were 84.0 μg/m^3^, 60.1 μg/m^3^, 25.1 μg/m^3^ and 41.7 μg/m^3^, respectively) affected YLL of old death cases more than that of young death cases. If true, this highlights the significance to public health of controlling air pollution timely when it is at a relatively low level.

We observed a trend towards a stronger association of gaseous pollutants (SO_2_ and NO_2_) with the outcomes in females but a stronger association of particulate matter (PM_10_ and PM_2.5_) with the outcomes in males. These were opposite to the results of Guo *et al.*^16^, in which the association of daily YLL with gaseous pollutants was stronger in males but that with particulate matter was stronger in females. The mechanism behind the discrepancy remains to be clarified. In fact, previous studies have yielded inconsistent results on the impact of air pollution in relation to sex. For example, some studies suggested a stronger association of PM_10_ with mortality in males^20^, while others reported that females were more vulnerable to air pollution because of their smaller airways and greater airway reactivity than those of males^21^,^22^. Non-biological factors such as lower socioeconomic status, education and poorer working conditions might also contribute to greater vulnerability of females^23^.

In this study, an increase of 10-μg/m^3^ in PM_10_, PM_2.5_, SO_2_ and NO_2_ was associated with 4.27, 2.97, 29.98 and 16.58 YLL, and 0.53%, 0.57%, 2.89% and 1.65% increase in daily death counts, respectively. In addition, the associations of PM with outcomes became weaker after gaseous pollutants, SO_2_ in particular, were included in the model. The estimates for associations with NO_2_ also decreased when SO_2_ was added, while inclusion of the other three pollutants into the model did not influence the estimates for association with SO_2_ much. The relative impact of PM versus gaseous pollutant and that of SO_2_ versus NO_2_ as shown by these results differ from some previous studies^10^,^24^,^25^. For example, the study of Guo *et al.* based on data of Beijing found that the impact of PM was more significant than that of SO_2_ and NO_2_. The CAPES study, which covered 17 Chinese cities other than Ningbo, showed that a 10-μg/m^3^ increase of two-day moving averaged SO_2_ and NO_2_ was associated with 0.75% and 1.63% increase of daily death counts^24^,^25^. However, there is actually no consensus on the relative impact of these pollutants. For example, a systematic review of 33 Chinese studies reported that the impact of SO_2_ and NO_2_ was more significant than that of PM^10^, and Guo *et al.* found that the impact of SO_2_ was larger than that of and NO_2_, both studies supporting our results. The difference between previous studies and the present one might be attributable to the differences in the concentrations of other pollutants such as O^3^, local meteorological conditions and demographic characteristics of different populations^17^,^18^,^19^.

Another potential reason behind the relatively stronger impact of SO_2_ in Ningbo might have to do with its relatively low concentration as compared with that in many other cities of China. Studies have shown that the concentration-response relationship between SO_2_ and mortality was steeper at low concentrations than at higher concentrations of SO_2_ 26, which means that in certain scenarios the association of SO_2_ with mortality might strengthen as SO_2_ concentration declined. A study conducted in Hong Kong has found that although the level of SO_2_ decreased substantially to a mean level of about 15 μg/m^3^, it remained most consistently associated with mortality^27^. This further supports the abovementioned notion that timely air pollution control when the pollution is at a relatively low level is very important. In addition, the finding indicates that stricter standards for air pollutants might need to be set and implemented in China for the sake of public health. This finding also lends support to the “controlling/reducing total SO_2_ emissions” strategy currently adopted by the government^24^.

Previous studies have investigated the modifying effects of weather conditions on the association between air pollution and mortality, with inconsistent results. For example, some studies found that the pollutant-mortality association was stronger in warm days in the northern regions of the USA, Brisbane of Australia, Seoul of South Korea, and Tianjin of China^28^,^29^,^30^,^31^, while others found the association stronger in cool season in Beijing, Shanghai, Wuhan, and Hong Kong of China^11^,^12^,^27^,^32^,^33^,^34^,^35^. In Ningbo, our study found that the adverse health effects of air pollution were much stronger in warm days with high temperature and low humidity than in others. Although not fully understood, this might be explained in part by the following reasons. First, the components of air pollution in Ningbo might vary across seasons, with the most toxic mixture of pollutants having a maximum in warm months^28^. Second, the stronger effects of air pollution in warm days might well be a result of more time people spent outdoors and more exposure to the pollution^28^. Third, high humidity might help reduce the particles in the air and protect the mucous membranes against toxic irritants^11^, while low humidity might interact with air pollution in an opposite way. The difference in the modifying effects of temperature and humidity in different cities might be related to various reasons such as different latitude, weather conditions, and the prevalence of air-conditioning system^12^.

The present study has several strengths. First, different from most previous studies, it used YLL as a key outcome to measure the impact of air pollution on premature deaths, which is generally more straightforward and accurate than daily death counts^13^,^14^,^16^. This allows people to better understand the burden of air pollution on population health. Second, as the air pollution level in Ningbo is close to the World Health Organization standards or Chinese Grade-II standards^3^,^36^, this study allows a better understanding of the adverse health impact of relatively low-level air pollution, which could differ from those in the high-pollution-level and heavily studied areas in China, such as Beijing^10^,^16^. Indeed, this study yielded some results different from previous studies^16^ and thus provides a unique evidence basis for the policy-making in air pollution control of China.

However, this study also has some limitations. First, as with most studies on air pollution, the exposure to air pollution was measured at population rather than individual level, and individual risk factors for mortality such as smoking, drinking and underlying diseases were unknown and uncontrolled in the analyses. Thus, we could not exclude the possibility of ecological bias and confounding effects of other mortality risk factors. Second, in this study, YLL was determined on the basis of life expectancy of the general population in China, which might overestimate YLL to some degree. This is because many death cases might have various underlying diseases, and their life expectancy at the age of death was shorter than that of the general population at the same age^13^. Third, as this study was based on the data from Ningbo alone and the association with air pollution may vary across different cities for multiple reasons, generalization of the results of this study should take local conditions into account.

In conclusion, we found significant associations of PM_10_, PM_2.5_, SO_2_ and NO_2_ with daily YLL and daily death counts in Ningbo, China, and the associations with both outcomes were more evident in the elderly. These findings highlight the importance and urgency of air pollution control. They also suggest that stricter standards for air pollutants might need to be set and implemented in China.

## Methods

### Population, mortality and YLL data

Ningbo is a port city located in the eastern part of Zhejiang Province (Fig. 1), with a high level of economic development comparing to the general situation of China (Gross Domestic Product per capita in 2013: United States dollars 15,046 vs 6,767)^37^,^38^. The number of registered residents of Ningbo was 5.8 million in 2013, distributed in an area of approximately 9,816 square kilometers^38^.

For this study, anonymous data on non-accidental deaths between January 1, 2009 and December 31, 2013 were obtained from local mortality register based in the Ningbo Municipal Center for Disease Prevention and Control, which was restricted to registered residents only and included 163,704 non-accidental deaths (92,314 male, 71,390 female) for the five study years. The mortality data were coded according to the 10^th^ revision of the International Statistical Classification of Diseases and Related Health Problems^39^, with the sex and age at death among other variables documented as well.

Daily death count was the number of deaths occurring on a single day. YLL of each death case was equal to the sex- and age-group-specific life expectancy of Chinese population in 2012, as estimated by the World Health Organization^40^. Daily YLL was calculated by summing the YLL of all the death cases who died on a same single day. Both daily death counts and daily YLL were stratified by sex (male vs female), age (≤65 years vs >65 years), and cause of death (respiratory vs circulatory vs others).

### Exposure assessment

The concentrations of PM_10_, PM_2.5_, NO_2_ and SO_2_ were monitored consecutively at 11 monitoring sites that cover all districts and counties of Ningbo, including both urban and suburban areas. The pollutants concentrations were measured according to the Chinese National Ambient Air Quality Standard^36^. The Environment Monitoring Center of Ningbo collects the pollutants data from the monitoring sites and records the concentrations on an hourly basis, from which the daily average concentrations of pollutants for individual monitoring sites and then the average levels for the whole Ningbo city were derived. Meteorological data, including daily mean temperature, relative humidity, air pressure and wind speed, were obtained from the China Meteorological Data Sharing Service System. Data for PM_2.5_ were available from 2011 to 2013 only, while data for the other pollutants and all meteorological parameters covers all the five study years, i.e. 2009–2013.

### Statistical analysis

As YLL in this study follows a normal distribution (Supplementary Figure S1), and previous studies have shown that the association between air pollution and YLL was approximately linear^16^, we used linear regression with a distributed lag model (DLM) to estimate the impact of air pollutants on YLL, which can be written as


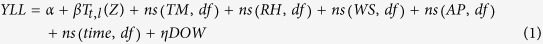


where *t* is the day of observation; *l* is the number of lag days; *Z* represents individual air pollutants; *T*_*t,l*_ is a matrix obtained by applying the DLM to air pollutants; and *β* is the vector of coefficients for *T*_*t,l*_; We employed a linear function to estimate the linear association and a natural cubic spline function to estimate the so-called “lag effects” of air pollutants. Degrees of freedom for the lag structure were chosen based on Akaike Information Criterion^41^. In this study, it was found that 5 degrees of freedom best fit the model. The function ns() is a natural spline. *TM* represents temperature; *RH* represents relative humidity; *WS* represents wind speed; and *AP* represents air pressure. Degrees of freedom for these meteorological variables were set to 3 in accordance with previous studies^42^,^43^,^44^. The variable “time” with a value ranging from 1 to 1,826 is the sequence number of individual days of the five study years listed in temporal order, with seven degrees of freedom per year to control for secular trend and seasonality. *DOW* represents day of week, which was used as a dummy variable, and 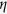 is a vector of coefficients.

The “lag effects” of air pollution were first examined using a single day lag (from lag 0 to lag 10), based on which the most likely lag days were determined and the cumulative association with air pollution over the lag period was estimated. The changes in daily YLL associated with a 10-μg/m^3^ increase in air pollutants were estimated. The correlation between air pollutants and meteorological conditions was examined by the spearman correlation function. Single-pollutant model was used to examine the main association of each pollutant with daily YLL, while two- and three-pollutant models were used to examine the stability of these associations. The effect estimates from the two- or three-pollutant model were not the sum of the effects of different pollutants, but rather referred to the independent effect of a certain pollutant after adjusting for the potential confounding caused by other pollutants.

To evaluate the impact of air pollutants on daily death counts and then compare it with that on YLL, the following regression model was used, with daily count of deaths as the dependent variable following a Poisson distribution.


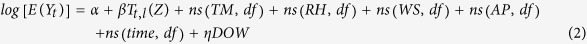


*E(Y*_*t*_) is the expected deaths count on day *t*, while the independent variables, lag structure and relevant degrees of freedom in this model are similar to those in the YLL model as expressed in equation (1). The regression model was used by previously published studies^44^.

An autocorrelation function (i.e. *pacf* function) was employed to check the fitness of the above models and if seasonality and autocorrelation had been successfully removed by examining the residuals over time. As data for PM_2.5_ were available from 2011 to 2013 only, we conducted sensitivity analyses by using data of the three years alone to see whether the main results for the other pollutants would significantly change. Additional sensitivity analyses were conducted by changing the degrees of freedom per year of time from 6 to 8. Stratified analyses were conducted according to sex, age and cause of death to identify populations who were potentially more sensitive to air pollution. As previous studies found that temperature and humidity may modify the effects of air pollution^11^,^12^, we also conducted stratified analyses according to the combination of temperature and humidity (high temperature and high humidity; high temperature and low humidity; low temperature and high humidity; low temperature and low humidity) to demonstrate potential effect modification. To maintain statistical power in stratified analyses, the cut-offs used to divide temperature or humidity into “high” or “low” level was their median values rather than 25 percentile, 75 percentile, or other point values. All statistical analyses were conducted with R software, version 2.15.0, using the dlnm and spline packages^45^,^46^.

## Additional Information

**How to cite this article**: He, T. *et al.* Ambient air pollution and years of life lost in Ningbo, China. *Sci. Rep.* **6**, 22485; doi: 10.1038/srep22485 (2016).

## Supplementary Material

Supplementary Information

## Figures and Tables

**Table 1:** Daily air pollutants, meteorological conditions, years of life lost and death counts in Ningbo, China, 2009–2013*.

	Minimum	25% quartile	Median	75% quartile	Maximum	Mean	Standard deviation
PM_10_ (μg/m^3^)	13	50	71	101	660	84.0	53.5
PM_2.5_ (μg/m^3^)	7	34	51	75	457	60.1	40.2
SO_2_ (μg/m^3^)	4	14	20	32	108	25.1	15.2
NO_2_ (μg/m^3^)	7	28	39	53	132	41.7	19.3
Temperature (°C)	−2.2	9.7	18.7	25.3	34.4	17.6	9.1
Relative humidity (%)	19	65	73	81	95	71.9	11.9
Air pressure (Pa)	985.9	1008.1	1015.5	1022.2	1037.3	1015.5	8.7
Wind speed (m/s)	0.0	1.0	1.6	2.5	6.6	1.8	1.1
Years of life lost (years)							
Total	573	936	1058	1204	1926	1078	198.0
Men	270	544	630	730	1204	639.4	136.9
Women	132	357	430	511	957	439.1	114.3
Age ≤65 years	178	446	538	634	1021	544.6	140.1
Age >65 years	201	448	520	610	1004	533.8	115.8
Respiratory deaths	18	76	103	139	365	111	49.8
Cardiovascular deaths	67	196	245	299	544	253	76.5
Deaths from other causes	331	613	707	810	1248	714.5	143.7
Daily death counts (No. of deaths)							
Total	45	77	87	101	166	89.7	17.9
Men	24	43	49	57	89	50.6	10.5
Women	15	32	38	45	78	39.1	9.9
Age ≤65 years	6	16	20	23	35	19.7	4.7
Age >65 years	29	58	67	80	136	70.0	16.3
Respiratory deaths	2	11	15	20	45	15.8	6.3
Cardiovascular deaths	8	21	26	32	60	27.0	7.9
Deaths from other causes	23	41	46	52	77	46.8	8.2

*The data on PM_2.5_ was collected from 2011 to 2013 only.

**Table 2:** Spearman correlation between air pollutants and meteorological conditions in Ningbo, China, 2009–2013*.

	PM_10_	PM_2.5_	SO_2_	NO_2_	Mean temperature	Relative humidity	Air pressure	Wind speed
PM_10_	1.00	0.85	0.72	0.73	−0.41	−0.34	0.41	−0.27
PM_2.5_	—	1.00	0.71	0.72	−0.49	−0.21	0.45	−0.32
SO_2_	—	—	1.00	0.80	−0.65	−0.33	0.64	−0.22
NO_2_	—	—	—	1.00	−0.64	−0.11	0.60	−0.33
Mean temperature	—	—	—	—	1.00	0.03	−0.89	0.07
Relative humidity	—	—	—	—	—	1.00	−0.17	−0.22
Air pressure	—	—	—	—	—	—	1.00	−0.08
Wind speed	—	—	—	—	—	—	—	1.00

*The analyses for PM_2.5_ were based on data collected from 2011 to 2013 only. All correlation coefficients not equal to 1 were statistically significant, with P<0.01.

**Table 3:** Association of a 10-μg/m^3^ increase of air pollutants (lag 0–3 day) with years of life lost and non-accidental deaths in Ningbo, China, 2009–13, using single-, two- and three-pollutant models*.

Pollutant and model	Years of life lost (95% CI)	Percentage increase in death (95% CI)
PM_10_		
Single-model	4.27 (1.17 to 7.38)	0.53 (0.29 to 0.76)
+SO_2_	−1.99 (−6.12 to 2.14)	−0.09 (−0.40 to 0.23)
+NO_2_	1.90 (−1.95 to 5.74)	0.29 (−0.01 to 0.58)
+SO_2_+NO_2_	−1.52 (−5.76 to 2.72)	−0.03 (−0.35 to 0.29)
PM_2.5_		
Single-model	2.97 (−2.01 to 7.95)	0.57 (0.20 to 0.95)
+SO_2_	−4.52 (−11.00 to 1.96)	−0.01 (−0.50 to 0.48)
+NO_2_	−1.13 (−7.22 to 4.96)	0.23 (−0.23 to 0.69)
+SO_2_+NO_2_	−4.38 (−11.06 to 2.31)	0.01 (−0.50 to 0.51)
SO_2_		
Single-model	29.98 (19.21 to 40.76)	2.89 (2.04 to 3.76)
+PM_10_	32.73 (19.20 to 46.27)	2.77 (1.72 to 3.83)
+NO_2_	29.78 (13.42 to 46.14)	2.99 (1.72 to 4.29)
+PM_10_+NO_2_	30.85 (13.68 to 48.03)	2.84 (1.51 to 4.18)
NO_2_		
Single-model	16.58 (8.19 to 24.97)	1.65 (1.01 to 2.30)
+PM_10_	14.78 (4.33 to 25.23)	1.35 (0.56 to 2.15)
+SO_2_	−1.94 (−14.53 to 10.66)	−0.25 (−1.20 to 0.71)
+PM_10_+SO_2_	0.10 (−13.05 to 13.24)	−0.22 (−1.21 to 0.79)

*The analyses are adjusted for seasonality, day of the week, temperature, relative humidity, air pressure and wind speed. The analyses for PM_2.5_ were based on data collected from 2011 to 2013 only.

**Table 4:** Association of a 10-μg/m^3^ increase of air pollutants (lag 0–3 day) with increase of years of life lost and non-accidental deaths in Ningbo, China, 2009–2013, using single-pollutant models, according to sex, age and cause of death*.

Outcome & Pollutant	Sex		Age		Cause of death		
	Male	Female	≤65 years	>65 years	Respiratory	Cardiovascular	Others
Increase of years of life lost (95% CI)							
_ _PM_10_	2.33 (−0.01 to 4.68)	1.94 (0.02 to 3.86)	1.32 (−1.25 to 3.88)	2.96 (1.40 to 4.52)	0.74 (0.00 to 1.48)	0.19 (−1.01 to 1.39)	3.35 (0.76 to 5.93)
_ _PM_2.5_	2.26 (−1.61 to 6.13)	0.71 (−2.36 to 3.79)	−1.18 (−5.34 to 2.98)	4.15 (1.62 to 6.68)	1.48 (0.29 to 2.67)	0.22 (−1.80 to 2.24)	1.27 (−2.90 to 5.44)
_ _SO_2_	12.79 (4.62 to 20.97)	17.19 (10.55 to 23.83)	14.23 (5.31 to 23.15)	15.75 (10.34 to 21.16)	4.31 (1.74 to 6.88)	7.74 (3.57 to 11.9)	17.93 (8.91 to 26.94)
_ _NO_2_	7.90 (1.57 to 14.24)	8.67 (3.51 to 13.85)	7.33 (0.41 to 14.25)	9.25 (5.04 to 13.46)	1.12 (−0.87 to 3.12)	3.57 (0.33 to 6.81)	11.89 (4.90 to 18.88)
Percentage increase in death (95% CI)							
_ _PM_10_	0.53 (0.24 to 0.82)	0.52 (0.19 to 0.86)	0.30 (−0.13 to 0.73)	0.58 (0.32 to 0.85)	0.86 (0.38 to 1.36)	0.43 (0.05 to 0.81)	0.47 (0.18 to 0.76)
_ _PM_2.5_	0.63 (0.15 to 1.10)	0.50 (−0.02 to 1.03)	−0.03 (−0.73 to 0.67)	0.73 (0.31 to 1.15)	0.99 (0.19 to 1.79)	0.46 (−0.19 to 1.11)	0.08 (−0.06 to 0.23)
_ _SO_2_	2.38 (1.33 to 3.45)	3.55 (2.34 to 4.78)	2.37 (0.79 to 3.98)	3.04 (2.08 to 4.01)	4.03 (2.28 to 5.82)	2.78 (1.40 to 4.17)	2.58 (1.51 to 3.65)
_ _NO_2_	1.21 (0.43 to 2.01)	2.21 (1.31 to 3.13)	1.40 (0.23 to 2.58)	1.72 (1.00 to 2.45)	1.77 (0.44 to 3.11)	1.69 (0.66 to 2.73)	1.61 (0.83 to 2.40)

*The analyses are adjusted for seasonality, day of the week, temperature, relative humidity, air pressure and wind speed. The analyses for PM_2.5_ were based on data collected from 2011 to 2013 only.

**Table 5:** Modifying effect of temperature and relative humidity level on years of life lost and daily number of deaths for a 10-μg/m3 increment of pollutants*.

Outcome & Pollutant	Total	High temperature and high humidity	High temperature and low humidity	Low temperature and high humidity	Low temperature and low humidity
Increase of years of life lost (95% CI)					
_ _PM_10_	4.27 (1.17 to 7.38)	0.66 (−8.85 to 10.17)	16.51 (7.94 to 25.07)	3.08 (−3.38 to 9.22)	4.82 (−1.14 to 10.79)
_ _PM_2.5_	2.97 (−2.01 to 7.95)	1.08 (−13.44 to 15.60)	12.10 (−1.96 to 26.16)	8.98 (−2.06 to 20.03)	3.00 (−7.02 to 13.03)
_ _SO_2_	29.98 (19.21 to 40.76)	21.52 (−32.45 to 75.49)	48.29 (5.83 to 90.75)	39.56 (18.34 to 60.78)	26.56 (7.97 to 45.15)
_ _NO_2_	16.58 (8.19 to 24.97)	28.63 (4.82 to 52.44)	40.49 (16.33 to 64.66)	23.99 (7.65 to 40.33)	20.37 (4.87 to 35.87)
Percentage increase in death (95% CI)					
_ _PM_10_	0.53 (0.29 to 0.76)	0.89 (0.04 to 1.75)	1.78 (1.06 to 2.50)	0.49 (0.00 to 0.98)	0.81 (0.32 to 1.30)
_ _PM_2.5_	0.57 (0.20 to 0.95)	1.14 (−0.14 to 2.43)	1.78 (0.61 to 2.96)	1.28 (0.46 to 2.11)	0.41 (−0.43 to 1.27)
_ _SO_2_	2.89 (2.04 to 3.76)	7.23 (2.44 to 12.23)	6.01 (2.34 to 9.81)	4.16 (2.59 to 5.75)	3.07 (1.59 to 4.56)
_ _NO_2_	1.65 (1.01 to 2.30)	3.12 (0.95 to 5.34)	4.04 (1.98 to 6.15)	2.73 (1.51 to 3.96)	2.43 (1.16 to 3.71)

*The analyses are adjusted for seasonality, day of the week, air pressure and wind speed. The analyses for PM_2.5_ were based on data collected from 2011 to 2013 only.
