# Supplementary material for: Ambient air pollution and years of life lost in Ningbo, China
Source: Sci Rep. 2016 Mar 1;6:22485. doi: 10.1038/srep22485 (PMC4772387; doi:10.1038/srep22485)
Supplement: Supplementary Information [file srep22485-s1.pdf]

Title

Ambient air pollution and years of life lost in Ningbo, China

Author list and affiliations

Tianfeng He[1,*], Zuyao Yang[2,*], Tao Liu[3], Yueping Shen[4], Xiaohong Fu[2], Xujun Qian[4], Yuelun Zhang[2], Yong Wang[1], Zhiwei Xu[5], Shankuan Zhu[6], Chen Mao[2,7], Guozhang Xu[1,†], Jinling Tang[2,7,†]

[1] Ningbo Center for Disease Control and Prevention, Ningbo, China

[2] Division of Epidemiology, JC School of Public Health and Primary Care, The Chinese University of Hong Kong, Hong Kong

[3] Guangdong Provincial Institute of Public Health, Guangdong Provincial Center for Disease Control and Prevention, Guangzhou, China

[4] School of Public Health, Soochow University, Suzhou, China

[5] School of Public Health and Social Work, Queensland University of Technology, Brisbane, Australia

[6] Injury Control Research Center, Zhejiang University School of Public Health, Hangzhou, China

[7] Shenzhen Key Laboratory for Health Risk Analysis, Shenzhen Research Institute of The Chinese University of Hong Kong, Shenzhen, Guangdong Province, China

* These authors contributed equally to the study.

† Corresponding authors: Prof. Jinling Tang, 4/F, School of Public Health Building, Prince of Wales Hospital, Shatin, New Territories, Hong Kong; phone: (+852) 2252 8779; fax: (+852) 2645 3098; email: jltang@cuhk.edu.hk. Professor Guozhang Xu, Ningbo Center for Disease Control and Prevention, No. 237 Yongfeng Road, Haishu District, Ningbo 315010, China; phone: (+86) 574 87273527; fax: (+86) 574 87361764; email: xugz@nbcdc.org.cn.

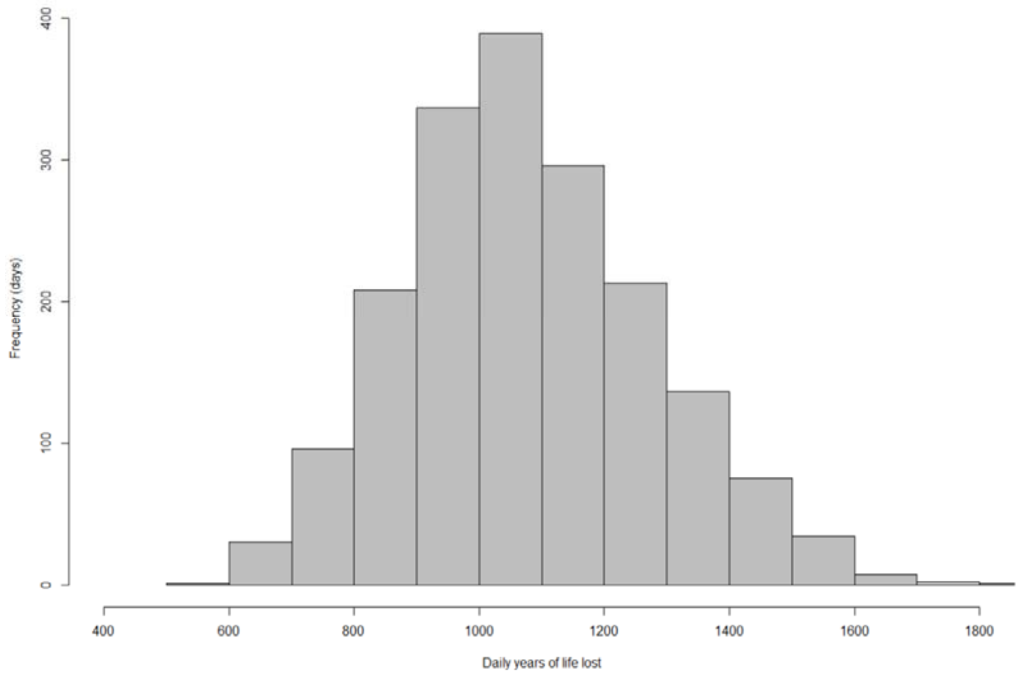

Supplementary Figure S1. Normal distribution of years of life lost

Supplementary Table S1. Association of a 10-μg/m^3 increase of air pollutants (lag 0-3 day) with years of life lost and non-accidental deaths in Ningbo, China, 2011-13, using single-, two- and three-pollutant models*

Pollutant and model	Years of life lost (95% CI)	Percentage increase in death (95% CI)
PM_{10}		
Single-model	5.77 (1.93 to 9.61)	0.66 (0.37 to 0.95)
$+SO_2$	0.96 (-4.34 to 6.26)	0.24 (-0.16 to 0.64)
$+NO_2$	4.10 (-0.70 to 8.89)	0.48 (0.12 to 0.84)
$+SO_2+NO_2$	1.18 (-4.30 to 6.65)	0.28 (-0.13 to 0.69)
$PM_{2.5}$		
Single-model	2.97 (-2.01 to 7.95)	0.57 (0.20 to 0.95)
$+SO_2$	-4.52 (-11.00 to 1.96)	-0.01 (-0.50 to 0.48)
$+NO_2$	-1.13 (-7.22 to 4.96)	0.23 (-0.23 to 0.69)
$+SO_2+NO_2$	-4.38 (-11.06 to 2.31)	0.01 (-0.50 to 0.51)
SO_2		
Single-model	25.07 (11.38 to 38.76)	2.44 (1.38 to 3.52)
$+PM_{10}$	22.26 (3.48 to 41.05)	1.92 (0.52 to 3.35)
$+PM_{2.5}$	32.51 (14.29 to 50.73)	2.43 (1.05 to 3.83)
$+NO_2$	21.15 (1.84 to 40.47)	2.17 (0.68 to 3.68)
$+PM_{10}+NO_2$	30.85 (13.68 to 48.03)	1.71 (0.07 to 3.38)
$+PM_{2.5}+NO_2$	17.31 (-4.33 to 38.94)	2.18 (0.55 to 3.84)
NO_2		
Single-model	14.56 (4.37 to 24.75)	1.51 (0.75 to 2.28)
$+PM_{10}$	8.89 (-3.88 to 21.66)	0.92 (-0.04 to 1.88)
$+PM_{2.5}$	15.36 (2.61 to 28.10)	1.23 (0.28 to 2.20)
$+SO_2$	2.17 (-12.13 to 16.47)	0.19 (-0.89 to 1.29)
$+PM_{10}+SO_2$	3.66 (-11.19 to 18.51)	0.11 (-1.01 to 1.25)
$+PM_{2.5}+SO_2$	4.90 (-9.95 to 19.75)	0.16 (-0.96 to 1.30)

* The analyses are adjusted for seasonality, day of the week, temperature, relative humidity, air pressure and wind speed. The analyses for $PM_{2.5}$ were based on data collected from 2011 to 2013 only.

Supplementary Table S2. Sensitivity Analyses: Association of a 10-μg/m^3 Increase of Air Pollutants (Lag 0-3 Day) With Increase of Years of Life Lost and Non-accidental Deaths in Ningbo, China, 2009-2013, Using Single-Pollutant Model With Different Degrees of Freedom (*df*) Per Year of Time*

Pollutant and *df*	Years of life lost (95% CI)	Percentage increase in death (95% CI)
PM_{10}		
df =7	4.27 (1.17 to 7.38)	0.53 (0.29 to 0.76)
df =6	4.21 (1.11 to 7.31)	0.51 (0.28 to 0.75)
df =8	4.09 (0.99 to 7.20)	0.52 (0.28 to 0.75)
$PM_{2.5}$		
df =7	2.97 (-2.01 to 7.95)	0.57 (0.20 to 0.95)
df =6	3.22 (-1.77 to 8.22)	0.61 (0.23 to 0.98)
df =8	2.98 (-2.02 to 7.98)	0.60 (0.23 to 0.97)
SO_2		
df =7	29.98 (19.21 to 40.76)	2.89 (2.04 to 3.76)
df =6	30.46 (19.79 to 41.14)	2.84 (1.99 to 3.69)
df =8	27.86 (16.96 to 38.75)	2.72 (1.86 to 3.58)
NO_2		
df =7	16.58 (8.19 to 24.97)	1.65 (1.01 to 2.30)
df =6	18.98 (10.78 to 27.17)	1.79 (1.16 to 2.42)
df =8	14.61 (6.17 to 23.05)	1.52 (0.88 to 2.16)

Abbreviations: PM_{10}, particulate matter with aerodynamic diameter <10μm; SO_2, sulfur dioxide; NO_2, nitrogen dioxide.

* The analyses are adjusted for seasonality, day of the week, temperature, relative humidity, air pressure and wind speed. The analyses for $PM_{2.5}$ were based on data collected from 2011 to 2013 only.
